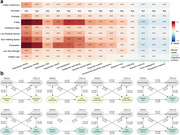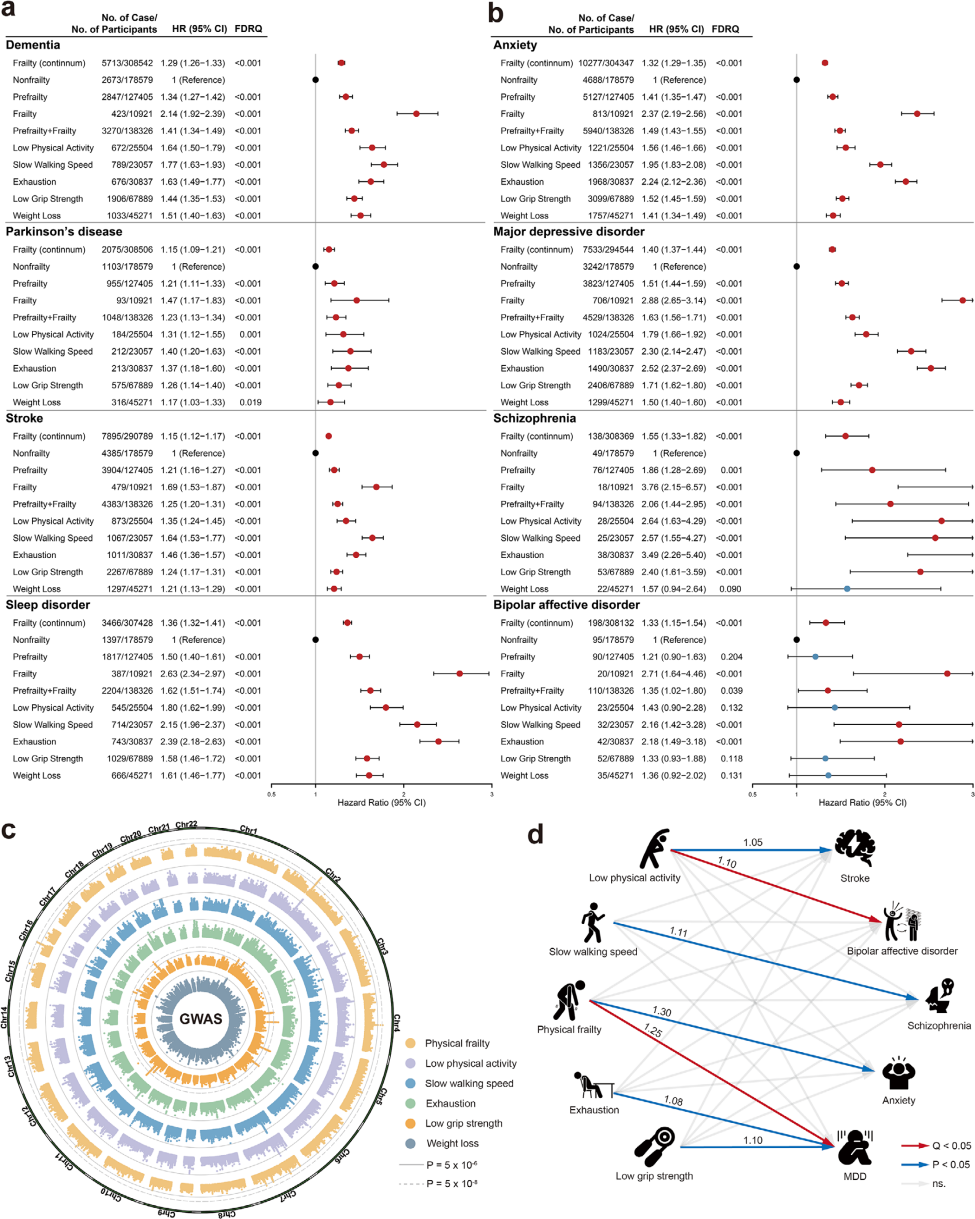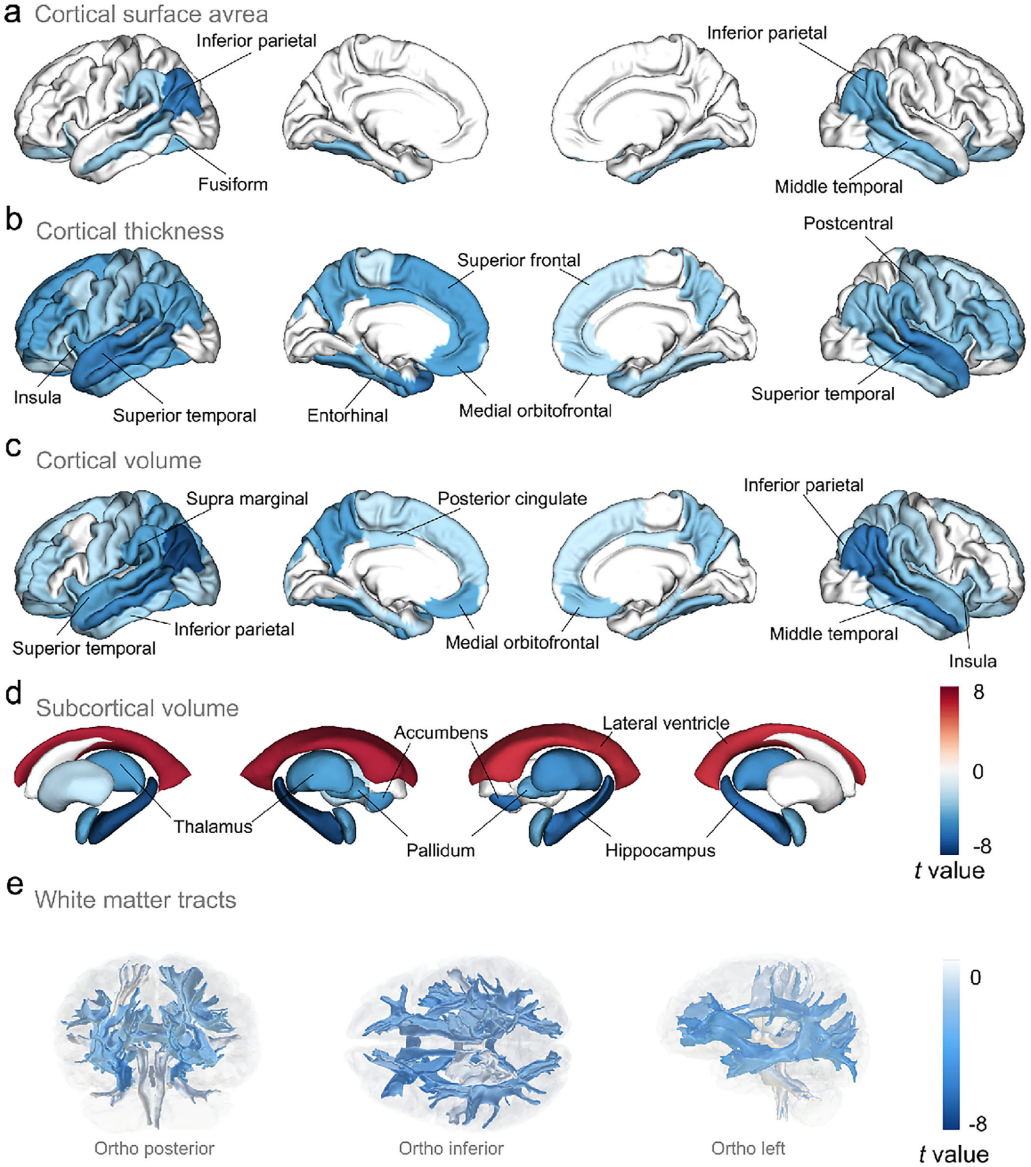# Physical frailty and brain health: a prospective cohort study

**DOI:** 10.1002/alz.093772

**Published:** 2025-01-09

**Authors:** Pei‐Yang Gao, Lan Tan, Jin‐Tai Yu

**Affiliations:** ^1^ Qingdao Municipal Hospital, Qingdao University, Qingdao China; ^2^ Qingdao Municipal hospital, Qingdao university, Qingdao, Shandong China; ^3^ Huashan Hospital, Fudan University, Shanghai, Shanghai China

## Abstract

**Background:**

Physical frailty is characterized as functional degeneration across multiple systems, which has been proposed as a risk factor for various health outcomes. However, the correlation between physical frailty and brain health, including brain function, brain disorders, and brain structure, remains unclear.

**Method:**

Here, 316905 participants from the UK Biobank were included in this prospective longitudinal study. Physical frailty was assessed by five phenotypes, including exhaustion, low grip strength, low physical activity, weight loss, and slow walking speed. Linear regression model and cross‐lagged panel model were firstly applied to evaluate the cross‐sectional and longitudinal relationship of physical frailty with brain function, including mental health and cognitive function. Cox proportional hazard model and Mendelian randomization analyses were further conducted to examine the longitudinal association and causal relationship between physical frailty and eight brain disorders. Finally, the cross‐sectional correlation between physical frailty and brain cortical and subcortical regions were assessed using linear regression model.

**Result:**

Over a median of 14.47 years follow‐up, 5943 (1.88%) new‐onset dementia were assessed. After adjusted covariates, a significant cross‐sectional and longitudinal relationship of physical frailty and five phenotypes with mental unhealth (four depression scales, life satisfaction, general mental status, and traumatic events) and worse cognition (reaction time, numeric memory, and fluid intelligence). Cox model and MR analyses revealed significant associations of physical frailty and five phenotypes with risk of incident dementia, Parkinson’s disease, stroke, sleep disorders, anxiety, major depressive disorder, and schizophrenia. Moreover, neuroimaging analyses emphasized the correlation of physical frailty with the cortical frontal cortex and subcortical regions including the thalamus, lateral ventricle, and hippocampus.

**Conclusion:**

This study provides evidence from multiple perspectives suggesting that physical frailty intervenes brain health and presents a strategy that early modification of physical frailty states may benefit the prevention of brain health.